# Risk factors associated with hepatitis B virus infection among pregnant women attending antenatal clinic at Felegehiwot referral hospital, Northwest Ethiopia, 2018: an institution based cross sectional study

**DOI:** 10.1186/s13104-019-4561-0

**Published:** 2019-08-15

**Authors:** Getnet Gedefaw, Fikadu Waltengus, Almaz Akililu, Kihinetu Gelaye

**Affiliations:** 1 Department of Midwifery, College of Health Sciences, Woldia University, Woldia, Ethiopia; 2 Department of Midwifery, College of Medicine and Health Sciences, Bahirdar University, Bahirdar, Ethiopia

**Keywords:** Pregnant women, Bahir Dar, Ethiopia, Hepatitis B virus

## Abstract

**Objective:**

This study aimed to determine the magnitude of serum HBsAg and the risk factors for hepatitis B virus infection among pregnant women in Bahir Dar. An institution based cross sectional study was implemented from February 1 to May 1, 2018 among 338 pregnant women attending antenatal care clinic at Felegehiwot referral hospital, Bahir Dar, 2018. Systematic random sampling technique was implemented. Blood sample was taken from 338 study participants and serum was tested for hepatitis B surface antigen (HBsAg) using Enzyme Linked ImmunoSorbent Assay.

**Results:**

The overall prevalence of hepatitis B virus infection among pregnant women were 16 (4.7%) (95% CI 2.7, 7.7). Having a history of blood transfusion (AOR = 5.2; 95% CI 1.2–22.3), having a history of multiple sexual partners (AOR = 4.6; 95% CI 1.1–19.6) and having a history tonsillectomy (traditional surgical procedure) (AOR = 3.4; 95% CI 1.1–10.1) were the significant risk factors for hepatitis B virus infection.

**Electronic supplementary material:**

The online version of this article (10.1186/s13104-019-4561-0) contains supplementary material, which is available to authorized users.

## Introduction

Hepatitis B virus (HBV) is a common cause of acute and chronic viral hepatitis worldwide. According to phylogenetic analyses, HBV can be classified into eight genotypes (A to H) based upon an inter-group divergence. Among these, genotypes B and C are most common among those with chronic HBV, while genotype A is most common among those with acute HBV. Hepatitis B virus (HBV) is DNA virus causing hepatitis in humans which is classified as chronic hepatitis B and acute hepatitis B virus infection. Acute hepatitis B in pregnancy is not associated with increased abortion rate, stillbirth, or congenital malformation but higher incidence of low birth weight was reported [1].

Hepatitis B virus (HBV) is transmitted by vertical transmission, between family members within households by contact of non-intact skin or mucous membrane with secreting or saliva containing, unsafe sexual intercourse, transfusion of HBV infected blood and blood products, perinatal transmission, horizontal transmission, nosocomial infection(commonly transmitted blood-borne virus in the healthcare setting),and percutaneous inoculation (contaminated medical equipment and sharing of contaminated syringes and needles among injecting drug users) [2]. Perinatal and early childhood transmissions are the main routes of HBV infection in endemic areas. The risk of HBV infection transmission decreases where there is periodic perinatal HBV screening, immunoprophylaxis given infants born with HBV infected mother and hepatitis vaccine given both to the high risk mother and the newborn. Therefore, administration of hepatitis B immunoglobulin (HBIG) in combination with hepatitis B vaccines as post exposure prophylaxis is very important since vertical transmission rate is nearly 100% [3]. Ethiopia is now identifying the magnitude of the problem and implementing the vaccination for health workers but still there is no accessibility and availability of vaccination of hepatitis B virus for those healthy mothers. Hence, this study aimed to determine the magnitude and associated factors of hepatitis B surface antigen virus among pregnant women attending antenatal care at Felegehiwot referral hospital, 2018.

## Main text

### Methods and materials

#### Study setting

Bahir Dar town is located in Amhara region, 565 km north-west of Addis Ababa. According to 2007 Ethiopian central statistical agency report, the total populations of Bahir Dar town administration is 221, 991. Of them 108,456 are males and 113,535 females. The hospital has different departments that provide specialized services in outpatient, inpatient and operation theatre departments. It provides services for approximately for more than 7 million people from the surrounding area. It has more than 415 beds and gives services for the western part of Amhara region as a referral hospital. There are more than 600 members of staff employed by the hospital and a further 200 employed by Bahir Dar university. Felegehiwot referral hospital provides care for the pregnant mothers widely in ANC, intrapartum and postpartum period. HBV screening is given for all pregnant mothers who had antenatal care follow up since it is one of the routine investigations during their first ANC follow up but free vaccination is not started yet.

#### Source population

All pregnant women who had antenatal care follow up at Felegehiwot referral hospital.

#### Study population

All pregnant women who were visited the antenatal care clinic during the first visit at Felegehiwot referral hospital.

##### Inclusion criteria

Women who had first antenatal care follow up at Felegehiwot hospital during the study period.

##### Exclusion criteria

Women whose antenatal care follow up were in other health institutions.

Women who referred from other health institutions.

Women who have been vaccinated HBV.

#### Study design and sample size determination

We designed an institutional based cross-sectional study to estimate magnitude of hepatitis B virus infection. The sample size was estimated using Epi Info 7 software using sample size determination for cross sectional studies. The parameters that were used to estimate the sample size were: confidence level of 95%, 5% margin of error and prevalence of outcome was 7.8%. The sample size was estimated by considering the prevalence of hepatitis B virus infection which was conducted in Hawassa hospital, Ethiopia. The sample size was 338 pregnant women.

#### Sampling techniques and procedures

Systematic random sampling technique was applied to select the study participants. We took 3 months’ average sampled population from registration book which was done at ANC clinic which is 626. To get kth interval = (source population) N/sample size (n_0_) = 626/338 ≈ 2. Then the first pregnant mother was randomly selected by lottery method. Then study participants were interviewed every two interval until the sample size was completed through systematic random sampling technique.

#### Data collection tools and procedures

Data collection was implemented both face to face interview through pretested structured questionnaire and chart review. A pre-tested structured questionnaire was consisting of socio-demographic and socioeconomic characteristics, risky socio cultural and behavioral factors, institution related factors and blood sample test was designed to collect patient serum hepatitis B surface antigen virus status by requesting laboratory investigation. One-day training was given for the supervisor and data collectors about data collection and sampling technique. There were two trained diploma midwives participated for data collection. Pre test was done to assess the content and face validity of the questionnaires. The investigator and supervisor made spot checking and reviewing the completed questionnaires on daily basis to ensure completeness and consistency of the information collected.

#### Laboratory methods

Blood sample was obtained from 338 pregnant women. A standard procedure was used to collect blood and process them for testing. All sera were screened for hepatitis B surface antigen (HBsAg) using Enzyme Linked Immunosorbent Assay (ELISA) (Hepanostika rapid kit test; Biomerieux, Boxtel, Netherlands with a sensitivity of 100% and specificity of 99.7%) in central laboratory which is found in Felegehiwot specialized Hospital compound.

#### Data processing and analysis

After declaring for completeness and consistency of the data, the data were entered into Epi Info version-7 and exported into SPSS version 23 statistical software for data cleaning, coded and analysis. Bivariate logistic regression analysis was done after dichotomizing the dependent variables. After checking associations of the variables, those with p < 0.2 in bivariate analysis was processed to multi-variable logistic regression analysis to control confounding factors. *p* value of < 0.05 was used to express the statistical significance of the variables.

### Results

#### Socio-demographic and socio economic characteristics

A total of 338 pregnant women were participating with a response rate of 100%. The mean age of the women was 26.84 years (ranged from 22 to 40 years) with a SD of ± 4.8 years. Two hundred sixty (76.9%) respondents were urban in residence. Regarding marital status, 327 (96.7) of them were getting married. 320 (94.6%) of the study participants had family history of HBV and 212 (62.7%) of them were multigravida women (Additional file 1: Table S1).

#### Risky socio cultural, behavioral and institution related factors

A total of 20 (5.9%) participants had been history of blood transfusion, while 92 (27.2%) had been hospitalized at some time during their lives. Among respondents 22 (6.5%) had a history of unprotected multiple sexual partners and 97 (28.7%) had a history of traditional tonsillectomy. No one was HIV positive among the study HBV positive participants (Table 1).

**Table 1:** Distribution of risky cultural, behavioral and institution related factors related to HBV infection among pregnant women attending antenatal care clinic at Felegehiwot referral Hospital from February to May, 2018, (n = 338)

Characteristics	HBV status of the mother		
	Frequency (%)	Positive (%)	Negative (%)
History of blood transfusions			
Yes	20 (5.9)	3 (15.0)	17 (85.0)
No	318 (94.1)	13 (4.1)	305 (95.9)
History of dental extraction			
Yes	30 (8.9)	1 (3.3)	229 (90.7)
No	308 (91.1)	15 (4.9)	293 (95.1)
History of surgical procedure			
Yes	41 (12.1)	1 (2.4)	40 (97.6)
No	297 (87.9)	15 (5.1)	282 (94.9)
History of abortion or miscarriage			
Yes	78 (23.1)	4 (5.1)	74 (94.9)
No	260 (76.9)	12 (4.6)	248 (95.4)
History of hospital admission			
Yes	92 (27.2)	4 (4.3)	88 (95.7)
No	246 (72.8)	12 (4.9)	234 (95.1)
History of circumcision/FGM			
Yes	283 (83.7)	13 (4.6)	270 (95.4)
No	55 (16.3)	3 (5.5)	52 (94.5)
Sharing of sharp materials			
Yes	32 (9.5)	3 (9.4)	29 (90.6)
No	306 (90.5)	13 (4.2)	293 (95.8)
History of unprotected multiple sexual partners			
Yes	22 (6.5)	3 (13.6)	19 (86.4)
No	316 (93.5)	13 (4.1)	303 (95.9)
History of unsafe injection of drugs			
Yes	5 (1.5)	1 (20.0)	4 (80.0)
No	333 (98.5)	15 (4.5)	318 (95.5)
History of body tattooed			
Yes	178 (52.7)	12 (6.7)	166 (93.3)
No	160 (47.3)	4 (2.5)	156 (97.5)
History of nose piercing			
Yes	10 (3)	0 (.0)	10 (100.0)
No	328 (97)	16 (4.9)	312 (95.1)
History of ear piercing			
Yes	320 (94.7)	16 (5.0)	304 (95.0)
No	18 (5.3)	0 (0.0)	18 (100.0)
History of traditional tonsillectomy			
Yes	97 (28.7)	10 (10.3)	87 (89.7)
No	241 (71.3)	6 (2.5)	235 (97.5)

#### Magnitude of HBV infection

A total of 16 (4.7%) with (95% CI 2.7, 7.7) respondents were found to be positive for HBV infection. A total of 8 (5.3%) respondent’s was observed in the age group of 26–30 years. Five (19.2%) respondents were reported in daily laborers. None of the respondents were positive among HBV vaccinated women (Table 2).

**Table 2:** Distribution of HBV among pregnant women attending antenatal clinic by socio demographic characteristics at Felegehiwot referral hospital, May 2018, (n = 338)

Characteristics	Frequency (%)	Status of HBV of the mothers	
		Positive N (%)	Negative (%)
Age (years)			
18–20	35 (10.4)	2 (5.7)	33 (94.3)
21–25	83 (24.6)	3 (3.6)	80 (96.4)
26–30	152 (45)	8 (5.3)	144 (94.7)
31–40	68 (20)	3 (4.4)	65 (95.6)
Residence			
Rural	78 (23.1)	7 (9.0)	71 (91.0)
Urban	260 (76.9)	9 (3.5)	251 (96.5)
Religion			
Orthodox	296 (87.6)	13 (4.4)	283 (95.6)
Muslim	32 (9.5)	3 (9.4)	29 (90.6)
Protestant	10 (3)	0 (0.0)	10 (100)
Marital status			
Single	1 (0.3)	0 (0.0)	1 (100)
Married	327 (96.7)	16 (4.9)	311 (95.1)
Divorced	10 (3.0)	0 (0.0)	10 (100)
Ethnicity			
Amhara	316 (93.5)	16 (5.1)	300 (94.9
Oromo	9 (2.7)	0 (0.0)	9 (100)
Tigre	7 (2.1)	0 (0.0)	7 (100)
Others	6 (1.8)	0 (0.0)	6 (100)
Occupation			
House wife	163 (48.2)	8 (4.9)	155 (95.1)
Employed	86 (25.4)	2 (2.3)	84 (97.7)
Merchant	57 (16.9)	1 (1.8)	56 (98.2)
Daily laborers	26 (7.7)	5 (19.2)	21 (80.8)
Student	6 (1.8)	0 (0.0)	6 (100)
Educational level			
No formal education	71 (21.0)	8 (11.3)	63 (88.7)
Primary	68 (20.1)	4 (5.9)	64 (94.1)
Secondary	82 (24.3)	1 (1.2)	81 (98.8)
College and above	117 (34.6)	3 (2.6)	114 (97.4)
Monthly income			
< 1000 ETB	20 (5.9)	1 (5.0)	19 (95.0)
1000–1500 ETB	32 (9.5)	3 (9.4)	29 (90.6)
1501–2300 ETB	34 (10.1)	1 (2.9)	33 (97.1)
> 2300 ETB	252 (74.5)	11 (4.4)	241 (95.6)
Previous place of birth			
Health facility	159 (47)	9 (5.7)	150 (94.3)
Home	52 (15.4)	4 (7.7)	48 (92.3)
TBA	0 (0.0)	0 (0.0)	0 (0.0)
No birth	127 (37.6)	3 (2.4)	124 (97.6)
No pregnancy			
Primigravida	104 (30.8)	2 (1.9)	102 (98.1)
Multigravida	212 (62.7)	12 (5.7)	200 (94.3)
Grand multigravida	22 (6.5)	2 (9.1)	20 (90.9)
HBV vaccination status			
Yes	17 (5)	0 (0.0)	17 (100)
No	321 (95)	16 (5.0)	305 (95)
Knowledge about way of transmission of HBV			
Yes	41 (12.1)	0 (0.0)	41 (100)
No	297 (87.9)	16 (5.4)	281 (94.6)

#### Risk factors associated with hepatitis B virus infection

Both bivariate and multivariable logistic regression analyses (backward conditional selection) were done to assess socio demographic and other predictable variables in relation to hepatitis B infection of the pregnant women. In bivariate analysis residency, educational status, previous place of birth, number of pregnancy, history of blood transfusion, history of body tattooing, having history multiple sexual partners, history of unsafe drug injection, history of sharing of sharp materials and the history of traditional tonsillectomy were factors significantly associated with hepatitis B infection.

Under multivariable logistic regression analysis showed that three predictor variables were statistically significant associated with HBV infections.

Pregnant women who had history of blood transfusion were 5.2 times more likely of being infected by HBV than pregnant women who had no history of blood transfusion [AOR = 5.2; 95% CI 1.2–22.3, p-value = 0.03].

Having a history of multiple sexual partners were 4.6 times more likely to be infected by HBV [AOR = 4.6; 95% CI 1.1–19.6, p-value = 0.04] than women who were living with their partner.

Pregnant women who had a history of traditional tonsillectomy were about 3.4 times more likely of being infected than those had no history of traditional tonsillectomy [AOR = 3.4; 95% CI 1.1–10.9, p-value = 0.03] (Table 3).

**Table 3:** Factors associated with HBV infection among pregnant women attending antenatal clinic at Felegehiwot referral hospital, Bahirdar, North West, Ethiopia, 2018, (n = 338)

Characteristics	HBV status of the mothers				p-value
	Positive %	Negative %	COR (95% CI)	AOR/ (95% CI)	
Residence					
Rural	7	71	0.4 (0.2, 1.1)	0.6 (0.2, 2.1)	0.34
Urban	9	251	1	1	
Educational level					
No formal education	8	63	0.2 (0.1, 0.9)	0.3 (0.1, 1.2)	0.09
Primary	4	64	0.5 (0.1, 1.2)	0.6 (0.1, 2.6)	0.42
Secondary	1	81	2.2 (0.3, 20.9)	2.6 (0.3, 23.2)	0.5
College and above	3	114	1	1	
Previous place of birth					
Health facility	9	150	0.8 (0.3, 2.5)	1.1 (0.2, 9.6)	0.98
Home	4	48	2.5 (0.7, 9.4)	1.5 (0.2, 19.8)	0.78
No birth	3	124	1	1	
No of pregnancy					
Primigravida	2	102	1	1	
Multigravida	12	200	0.33 (0.07, 1.49)	0.6 (0.2, 3.3)	0.53
Grand multigravida	2	20	0.20 (0.03, 1.48)	0.9 (0.1, 8.9)	0.89
History of blood transfusions					
Yes	3	17	4.2 (1.08, 16)	5.2 (1.2, 22.3)	0.03*
No	13	305	1	1	
History of sharing sharp materials					
Yes	3	29	2.4 (0.7, 8.7)	1.2 (0.3, 5.1)	0.81
No	13	293	1	1	
History of unprotected multiple sexual partners					
Yes	3	19	3.7 (1, 14.1)	4.6 (1.1, 19.6)	0.04*
No	13	303	1	1	
History of unsafe injection of drugs					
Yes	1	4	5.3 (0.6, 50.4)	6.5 (0.6, 76.8)	0.2
No	15	318	1	1	
History of body tattooed					
Yes	12	166	2.9 (1, 9)	1.6 (0.5, 6)	0.5
No	4	156	1	1	
History of traditional tonsillectomy					
Yes	10	87	4.5 (1.6, 12.8)	3.3 (1.1, 10.1)	0.03*
No	6	235	1	1	

* p < 0.05

### Discussion

Hepatitis B virus infection is a public health problem and a major cause of morbidity and mortality, particularly in developing countries [4].

In this study found to be that the magnitude of HBsAg among study participants were 16 (4.7%) (95% CI 2.7, 7.7) with a response rate of 100%. According to established criterion, the prevalence of HBsAg among pregnant women in this study area can be classified as an intermediate category [5].

This finding is in line with different studies were done with a proportion of 5.3% in Debre Tabor general hospital [6], 5.5% in Tigray [7], 3% St. Paul’s millennium medical college and Selam health center [8], 3.7% in Jimma [9], 6.9% in Deder hospital, eastern Ethiopia [10], 7.3% in Gondar health center [11], and 4.9% in Dessie Referral hospital [12]. This might be due to the sampling method, risky socio cultural and risky behavioral practice and methods used to screen HBsAg infection were the same.

But, relatively it is higher than 2.5% of prevalence which were reported from three public hospitals in Addis Ababa [13]. This difference might be due to risky socio cultural and behavioral practices were low. However, it is lower than the study conducted in Hawassa referral hospital 7.8% [14]. This difference might be due to the methods used to screen highly sensitive and specific and risky socio cultural and behavioral practiced were high.

However, higher results were reported in Mali 8% [15], Yemen 10.8% [16], Uganda 11.8% [17], Nigeria 12% [18] and Kenya 14.1 [19]. This variation might be due to differences in sampling method, geographical variation, cultural and behavioral differences regarding possible risk factors of HBV infection, and differences in the test methods employed to detect HBV infection.

Whereas, lower prevalence 0.14% to 0.97%, 0.9%, 1%, 1.5%, 1.6% and 2.1% were reported in USA [20], Brazil [21], Kenya [22], Libya [3], Saudi Arabia [23] and North Turkey [15], respectively. This variation may be due to in developed nations, where regular screening and vaccination for HBV were performed.

Having history of tonsillectomy (traditional surgical procedure) was an independent risk factor associated with HBV. This study finding is similar with the study done in different places of Ethiopia Deder [10], Dessie [12] and Nigeria [18]. This finding may be explained as this surgical procedure is performed via traditional manner where no sterilization technique used. Therefore, the virus is easily transmitted from the career to the healthy mother.

In this study having a history of blood transfusion was an independent risk factor for hepatitis B virus infection. The finding of this study is similar with the study done in Tanzania [24], Debre Markos, Northeast Ethiopia [25]. This is explained as due to the fact that hepatitis B virus is transmitted through any fluid/mucosal/blood contact from infected patients easily.

Women who had history of multiple sexual partners have high chance to be infected by hepatitis B virus infection than the counter parts. This study finding is consistent with the study done in northern Ethiopia [6], Deder [10], and Nigeria [12]. This finding may be explained as since hepatitis virus is blood born virus; blood, semen and other body fluids are primary source of infection that sexual contacts provide as mode of transmission.

### Conclusion

This study revealed that the magnitude of HBV infection was low. Having history of blood transfusion, having history of multiple sexual partners and having history of tonsillectomy (traditional surgical procedure) were significantly associated variables with HBV infection. Avoiding cultural malpractice, ensuring sterility while taking blood for transfusion and create awareness in the community about the transmission and prevention methods may decrease the magnitude of hepatitis B surface antigen virus infection.

## Limitation

This study was the inability to use more sensitive diagnostic methods like polymerase chain reactions, which would have help detecting occult HBV infection.

## Additional file

**Additional file 1: Table S1.** Socio demographic characteristics of pregnant women attending antenatal clinic at Felegehiwot referral hospital, May 2018 (n = 338).

## Data Availability

All related data has been presented within the manuscript. The data set supporting the conclusions of this article is available from the authors on request.

## Abbreviations

AIDS: Acquired Immunodeficiency Syndrome
ANC: Antenatal Clinic
AOR: adjusted odd ratio
APHI: Amhara public Health Institution
BSc: Bachelor of Science
CDC: Center for Disease Control
COR: crude odd ratio
DNA: deoxyribonucleic acid
ELISA: Enzyme Linked Immunosorbent Assay
FGM: female genital mutilation
HBeAg: hepatitis B “e” antigen
HBsAg: hepatitis B surface antigen
HBV: hepatitis B virus
HCV: hepatitis C virus
HIV: human immunodeficiency virus
IEC: Information Education Communication
MSc: Masters of Science
SPSS: Statistical Package for Social Science
USA: United States of America
WHO: World Health Organization
