# Supplementary material for: Risk factors associated with hepatitis B virus infection among pregnant women attending antenatal clinic at Felegehiwot referral hospital, Northwest Ethiopia, 2018: an institution based cross sectional study
Source: BMC Res Notes. 2019 Aug 15;12:509. doi: 10.1186/s13104-019-4561-0 (PMC6694615; doi:10.1186/s13104-019-4561-0)
Supplement: Supplementary file 1 — Additional file 1: Table S1. Socio demographic characteristics of pregnant women attending antenatal clinic at Felegehiwot referral hospital, May 2018 (n = 338). [file 13104_2019_4561_MOESM1_ESM.docx]

Table S1: Socio demographic characteristics of pregnant women attending antenatal clinic at Felegehiwot referral hospital, May 2018 (n=338).

| Characteristics | Frequency | Percentage (%) |
| --- | --- | --- |
| Age(yrs.)  18-20  21-25  26-30  31-40 |  |  |
|  | 35  83  152  68 | 10.4  24.6  45  20 |
| Residence  Rural  Urban | 78  260 | 23.1  76.9 |
| Religion  Orthodox  Muslim  Protestant | 296  32  10 | 87.5  9.5  3 |
| Marital status  Single  Married  Divorced | 1  327  10 | 0.3  96.7  3.0 |
| Ethnicity  Amhara  Oromo  Tigre  Others* | 316  9  7  6 | 93.5  2.7  2.1  1.7 |
| Educational level  No formal education  Primary  Secondary  College and above | 71  68  82  117 | 21.0  20.1  24.3  34.6 |
| Monthly income  <1000  100--1500  1501--2300  >2300 | 20  32  34  252 | 5.9  9.5  10.1  74.5 |
| Previous place of birth  Health facility  Home  TBA  No birth | 159  52  0  127 | 47  15.4  0  37.6 |
| Number of pregnancy  Primigravida  Multigravida  Grand multigravida | 104  212  22 | 30.8  62.7  6.5 |
| Family history of HBV  Yes  No  Unknown | 2  320  16 | 0.7  94.6  4.7 |
| HCV yes  No | 23  315 | 6.8  93.2 |
| Do you know about transmission of HBV?  Yes  No | 41  297 | 12.1  87.9 |

Others*(Benshangule Gumuz, Agew, Gurage)
